# A Clinical Experimental Model to Evaluate Analgesic Effect of Remote Ischemic Preconditioning in Acute Postoperative Pain

**DOI:** 10.1155/2016/5093870

**Published:** 2016-06-30

**Authors:** Francisco Elano Carvalho Pereira, Irene Lopes Mello, Fernando Heladio de Oliveira Medeiros Pimenta, Debora Maia Costa, Deysi Viviana Tenazoa Wong, Claudia Regina Fernandes, Roberto César Lima Junior, Josenília M. Alves Gomes

**Affiliations:** ^1^Surgery Department, Federal University of Ceará, Fortaleza, CE, Brazil; ^2^Walter Cantídio University Hospital, Fortaleza, CE, Brazil; ^3^Physiology and Pharmacology Department, Federal University of Ceará, Fortaleza, CE, Brazil

## Abstract

This study aims to evaluate the viability of a clinical model of remote ischemic preconditioning (RIPC) and its analgesic effects. It is a prospective study with twenty (20) patients randomly divided into two groups: control group and RIPC group. The opioid analgesics consumption in the postoperative period, the presence of secondary mechanical hyperalgesia, the scores of postoperative pain by visual analog scale, and the plasma levels interleukins (IL-6) were evaluated. The tourniquet applying after spinal anesthetic block was safe, producing no pain for all patients in the tourniquet group. The total dose of morphine consumption in 24 hours was significantly lower in RIPC group than in the control group (*p* = 0.0156). The intensity analysis of rest pain, pain during coughing and pain in deep breathing, showed that visual analogue scale (VAS) scores were significantly lower in RIPC group compared to the control group: *p* = 0.0087, 0.0119, and 0.0015, respectively. There were no differences between groups in the analysis of presence or absence of mechanical hyperalgesia (*p* = 0.0704) and in the serum levels of IL-6 dosage over time (*p* < 0.0001). This clinical model of remote ischemic preconditioning promoted satisfactory analgesia in patients undergoing conventional cholecystectomy, without changing serum levels of IL-6.

## 1. Introduction

Ischemic preconditioning (IPC) is defined as brief periods of ischemia, interspersed with reperfusion, prior to a sustained period of ischemia. This procedure is performed in order to prepare and protect the cell to the damage caused by a long period of ischemia [1]. It is a powerful innate mechanism of multiple organs protection which can be induced by transient occlusion of the blood flow of an organ. Recently, other functions, besides the protection from reperfusion injury, have been attributed to preconditioning, including promoting analgesia [2–4].

Many surgical and nonsurgical cardioprotective strategies have been developed to reduce the levels of ischemic injury, some more successful than others. In addition to its protective effects in ischemia-reperfusion injury, there is a considerable amount of evidence indicating the effects of IPC in inflammatory conditions of nonischemic nature, probably through a systemic action [5].

The effects of inflammatory cytokines have been demonstrated in relation to TNF-alpha, which has been identified as the major mediator involved in the development of tolerance to induced IPC [6]. A reduction was observed in IL-6 and IL-1*β* levels after limb ischemia in pigs [3] and increased IL-10 levels [7, 8]. Ischemic preconditioning has been proved to be beneficial in many clinical settings, most of them involving patients undergoing invasive or long duration procedures, which may result in a relative state of ischemia. Furthermore, there is evidence that this phenomenon has a chronic feature and can ensure protection and anti-ischemic inflammation in patients with metabolic syndrome, or cardiovascular and cerebrovascular diseases, or those at risk for recurrent ischemic attack [9].

The remote ischemic preconditioning (RIPC) is achieved by a series of short nonlethal ischemic periods, interspersed with periods of reperfusion in distant tissues, that results in a target organ protection in late ischemic events [10–13]. Kosieradzki (2002) [14] showed a decrease in proinflammatory expression of genes in leukocytes after PCIR induction. Kharbanda et al. (2001) [15] showed that the reduction in neutrophil migration and its activation plays a central role in the anti-inflammatory PCIR mechanism [3, 4, 16].

Postoperative pain has inflammatory and nociceptive nature. It results from the interaction between tissue damage and nociceptive sensory receptor stimulation through inflammatory mediators. Thus, considering that the mediators of inflammation are closely related to the reduction of the excitability threshold of the nociceptive primary afferent, and the release of factors related to the inflammatory response can be changed by RIPC, the hypothesis is reasonable that this simple procedure could affect the pain response, producing postoperative analgesia.

This study aims to evaluate the analgesic activity of a clinical model of ischemic preconditioning on postoperative pain resulting from subcostal incision, as well as the influence of this technique in the cytokines levels in the postoperative period.

## 2. Patients and Methods

### 2.1. Ethics

The study was approved by Walter Cantídio University Hospital's Scientific Ethical Committee, which is regularly affiliated to National Research Ethics Commission (CONEP) registered with number 015.03.12. The research was conducted in accordance with the Helsinki Declaration and the principles of Good Clinical Practice. The approval was ratified by the Department of Studies of Santa Casa de Misericordia Hospital in Fortaleza.

### 2.2. Study Design and Randomization

A randomized controlled trial, partially blind (evaluators of postoperative outcomes did not know the allocation group of patients), with prospective and quantitative nature was developed at Santa Casa de Misericordia Hospital from June 2013 to June 2014. After applying informed consent form, the patients were randomized utilizing sequentially numbered opaque sealed envelopes to one of two groups (ratio 1 : 1) by the study coordinator. The same anesthetic-surgical medical team did all procedures. All included patients were females, between the ages of 20 and 55 years, with cholelithiasis, physical status, ASA I or II (no comorbidities, or with 1 clinical morbidity well-defined and well controlled, according to criteria defined by American Society of Anesthesiology (ASA)). The exclusion criteria were patients under 20 or above 55 years old, those with signs or symptoms suggestive of acute cholecystitis or choledocholithiasis, and subjects with more than one comorbidity or with one comorbidity clinically uncontrolled or poorly defined. We also excluded those subjects who did not properly understood the methods assessment of postoperative pain, those who did not sign the informed consent form, and patients who presented hemodynamic changes or other serious intraoperative complications, as well as those who had an inappropriate spinal block.

### 2.3. Remote Ischemic Preconditioning

The choice of the experimental design was a challenge. There are no clinical studies using remote preconditioning for abdominal surgery. Thus, this model was based on previous studies using the technique in orthopedic surgery [2–4]. However, this technique had to be adapted. The remote ischemic preconditioning was induced by inflation of a pneumatic tourniquet in lower limb, located at the thigh. The reference value was 100 mmHg above systolic pressure, for a period of 5 min. The ischemia was preceded by the member elevation in 45 degrees to the gravitational drainage of blood during 3 minutes (partial exsanguination). Only one preconditioning cycle was performed after the anesthetic block. The interruption of blood flow and its return were documented using a pulse oximeter in the ipsilateral lower end [17].

### 2.4. Data Collection

Pain control in the postoperative period was indirectly assessed by measuring the intravenous morphine consumption during 24 hours. All patients received a predetermined and fixed analgesia with intravenous metamizole (1 g) every 6 hours. The visual analogue scale (VAS) was applied once 24 hours after surgery, asking about pain intensity in three situations: at rest, during deep inspiration, and during cough induction. Two independent investigators, blinded to the study group where the patient was allocated, performed all the postoperative VAS measurements.

**Figure 1:**
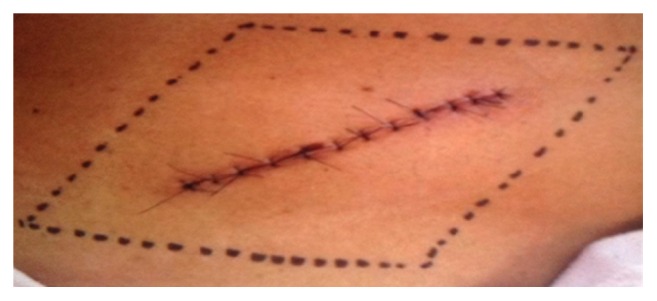
Hyperalgesia area.

**Figure 2:**
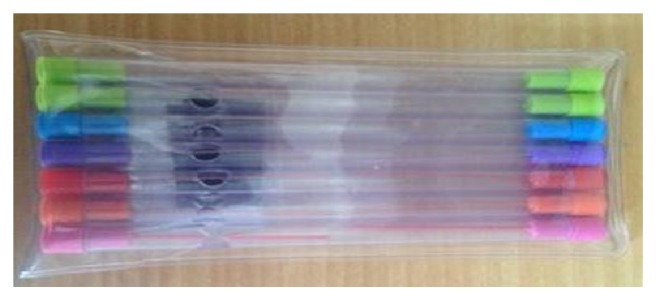
Von Frey filaments. Colors determine the variation of the force produced by applying the filament.

The presence of hyperalgesia produced by mechanical stimulation of the area surrounding the incision previously marked was observed 24 hours after the end of the procedure (Figure 1). This evaluation was according to the modified method described by Lavand'homme et al. [18] and based on the use of a sequence of Von Frey filaments (Figure 2) whose flexure strength produced pain in a range between 0.05 and 10 g. The stimulation was started on the borders of the investigated area towards the incision until the point where patient realized changing in the perception and started to describe it as pain, burning, and penetration. The value of the force, which produced the stimulus, was recorded. Values above 10 g were considered lack of hyperalgesia.

In order to assess the role of IL-6 in the RIPC effects on acute postoperative pain and hyperalgesia, 5 mL of blood samples was collected in four different moments: *T*0 (before ischemic preconditioning), *T*1 (30 min. after surgical incision), *T*2 (60 min. after surgical incision), and *T*3 (24 hours after the surgery).

### 2.5. Anesthetic-Surgical Procedure

The surgical technique was similar in all patients, performed by the same surgical team and based on classical technique performed for conventional surgery: right subcostal incision. Only three anesthesiologists, experienced in performing spinal blocks, participated in the implementation of standardized anesthesia with a spinal block in L2-L3 space using a needle gauge in the range 26-27, heavy bupivacaine 0.5%, for a total of 15 mg–20 mg (3-4 mL), and 5 micrograms of sufentanil.

### 2.6. Statistical Analysis of Data

Quantitative variables were initially analyzed by the Kolmogorov-Smirnov test to check the normality of distribution. For descriptive statistics, the mean and standard error of the continuous variables were calculated. Intergroup comparisons at each time point were performed by using the unpaired *t*-test (parametric data) and the Mann-Whitney test or Kruskal-Wallis (nonparametric variables). Qualitative variables were compared using the Fisher exact test [19]. In all analyses, the probability of error Type I (*α*) (level of significance) was set at 0.05 (5%) considering as statistically significant *p* value less than 0.05. The GraphPad PRISM® software version 5.00 for Windows (GraphPad Software, San Diego, California, USA, 2007) was used for both implementation of statistical procedures as for the preparation of graphics.

## 3. Results

There were no significant differences in the analysis of the variables: age, weight, duration of surgery, and ASA physical status between the two groups, as shown in Tables 1 and 2.

**Figure 3:**
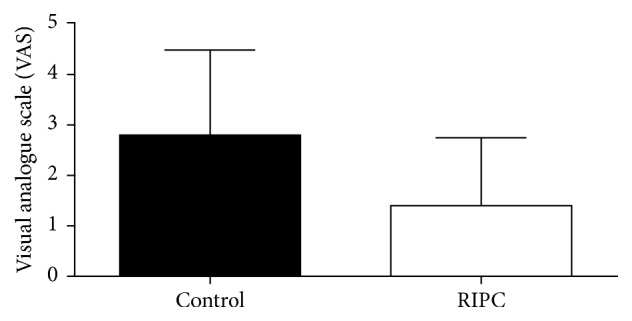
Morphine consumption. Values expressed as mean ± standard deviation of the mean. *p* = 0.0656. Analysis: KS test for normality of distribution followed by Mann-Whitney test. *p* values <0.05 were considered significant.

The overall total morphine consumption in 24 hours was significantly less in tourniquet group compared to the control group which can be shown in Figure 3.

**Figure 4:**
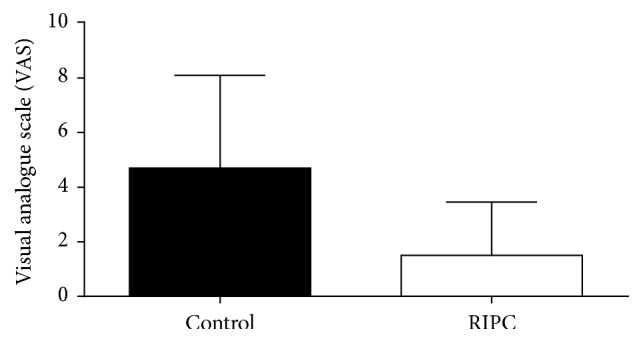
Intensity of postoperative pain at rest. Values expressed as mean ± standard deviation of the mean. *p* = 0.0087. Analysis: KS test for normality of distribution followed by Mann-Whitney test. *p* values <0.05 were considered significant.

**Figure 5:**
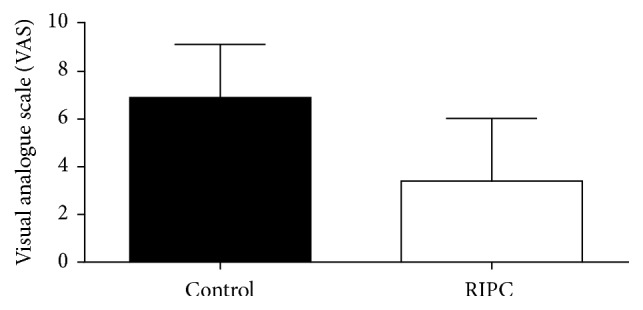
Intensity of postoperative pain on deep breathing. Values expressed as mean ± standard deviation of the mean. *p* = 0.0119. Analysis: KS test for normality of distribution followed by Mann-Whitney test. *p* values <0.05 were considered significant.

**Figure 6:**
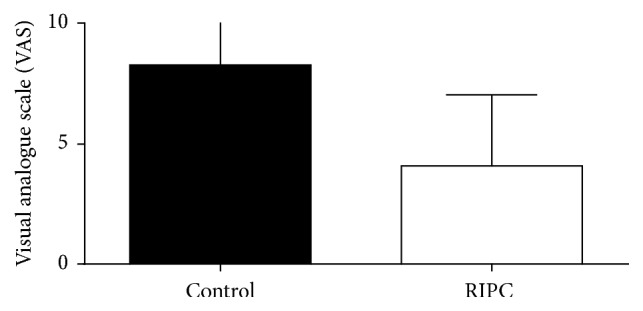
Intensity of postoperative pain in cough. Values expressed as mean ± standard deviation of the mean. *p* = 0.0015. Analysis: KS test for normal distribution followed by unpaired *t*-test. *p* values <0.05 were considered significant.

Analysis of the evaluation of the intensity of pain at rest and in deep breathing and coughing showed significantly lower VAS scores at Garrote group compared to the control group, as shown in Figures 4, 5, and 6.

**Figure 7:**
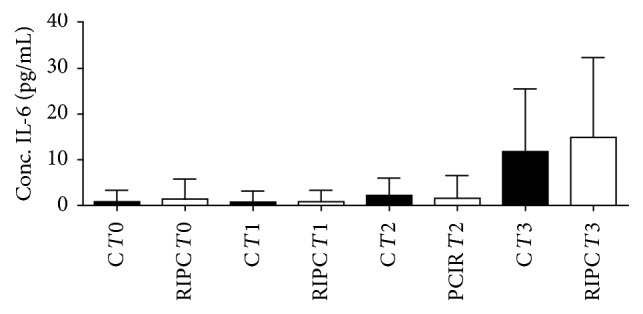
Values are expressed as mean + standard deviation of the average IL-6 concentration in the blood sample times (*T*0, *T*1, *T*2, and *T*3). *p* = 0.0001 for group comparison within the tourniquet over time and *p* = 0.779 for comparison between groups at the same time. *p* values <0.05 were considered significant. Analysis: KS test for normal distribution followed by Kruskal-Wallis test.

There were significant differences in IL-6 between the two dosage groupsin the course of time as shown in Figure 7 (*p* < 0.0001). There was a growing increase in the concentration of IL-6 with no difference statistically significant between the groups control and tourniquet in the times of each sample.

## 4. Discussion

The data presented in this study demonstrate a significant reduction in postoperative pain in patients undergoing conventional cholecystectomy who were submitted to remote preconditioning ischemic before the surgical procedure. It demonstrates an unprecedented way, the effect of remote ischemic preconditioning over abdominal surgery pain, since in previous publications, the pneumatic cuff was applied directly to the operated member [2–4].

During the past two decades, multiple variations on the theme of RIPC have been investigated, encompassing both in vitro and in vivo models. The cardioprotective effect has been the main focus; however RIPC protects the myocardium, but also other parenchymal organs and, notably, the vasculature [20]. Sousa Filho [21] working in an experimental model showed that RCPI exerts its antinociceptive action by inhibiting neutrophil migration. Although RIPC has been utilized in a number of clinical settings with promising results and many novel “downstream” mechanisms of RIPC have been discovered, its translation to pharmacological conditioning has not yet been convincingly demonstrated in clinical studies [22].

Studies where peripheral limb ischemia is the RPIC stimulus have mostly employed 3 or 4 episodes of 5 min arm and/or leg ischemia interspersed with 5 min reperfusion periods. However, these are empiric choices, the optimal algorithm has not been identified, and it has been postulated that “hyperconditioning” (i.e., an as-yet undefined, excessive number of conditioning episodes) may be deleterious [23]. Thus there is still no consensus about the time of tourniquet inflation enough to produce RICP. In this study, a single tourniquet inflation cycle was performed for five minutes that showed to be sufficient to obtain analgesic effects. Zarbock et al. [24] in a recent publication are questioning the need for intermittent tourniquet.

The laparoscopic cholecystectomy is considered the gold standard for the surgical treatment of gallstones and is replacing the conventional technique in most services [25]. However, in Brazil, there are large disparities in income distribution between regions and the health system underfunding meaning that, in many services, the only option remains the conventional technique with transverse subcostal incision resulting in significant postoperative pain with considerable increase in morbidity due to reduced lung expansion and cough with pain [26–28]. In these cases, the use of neuraxial block technique results in better pain control postoperatively when compared to general anesthesia alone [29–32]; however despite this evidence many patients receiving spinal block as anesthetic technique remain in pain postoperatively and are more susceptible to the risk of postoperative chronic pain [33, 34].

This study presents a clinical model of PCIR as a strategy that can be associated with other in the multimodal analgesia resulting in adequate pain control postoperatively [35] and meets a global trend of more effective guidelines settings for the treatment of postoperative pain [36, 37].

The higher reduction in morphine consumption postoperatively in RIPC group also needs to be presented as a very relevant finding, facing the possibility of reducing the complications arising from the adverse effects of opioids that increase postoperative morbidity [38].

An important question to be answered is how could PCIR act to produce analgesia? It has been demonstrated that circulating monocytes play a key role in ischemia/reperfusion injury RIPC downregulating the expression of a broad portfolio of proinflammatory genes in circulating monocytes [39] and reducing inflammatory circulating cytokines. However, in this study the serum dosage of interleukin 6 showed no reductions over time. Other researches confirmed similar results in healthy human volunteers subjected to RIPC where it was not associated with any difference in circulating markers of inflammation (e.g., interleukins 6, 8, and 10, or tumor necrosis factor levels) [40].

Details of the mechanisms for local release of the protective signal at the remote site and the contributions of neuronal and humoral pathways to the RPCI are not yet clear; however several extracellular signalling molecules, such as adenosine, bradykinin, and opioids, have been identified as mediators and effectors [41] supporting the evidence of opioid involvement contributing to the pharmacological preconditioning [42]. So, if RPCI releases endogenous opioids it would have an analgesic effect reducing morphine consumption in postoperative period.

Besides that, it seems that in most cases the common final step of the molecular mechanisms involved in preconditioning is the opening of potasium-ATP channels [43]. Recent studies indicate that PCI can increase the activity of the potasium-ATP channels and alleviate reperfusion injury of the myocardium through this mechanism [44, 45]. It is also known that the mechanism of action of opioids for analgesia production involves the opening of potassium channels in the postsynaptic neuron [46]. Based on these data, it is possible to suggest a synergistic mechanism between PCRI and opioid analgesic increasing the potency of morphine, explaining the lower level of pain and reduction in the consumption of morphine in the group subjected to PCRI.

In conclusion, this model of remote ischemic preconditioning is feasible to reproduce in clinical setting, improved pain control, and reduced morphine use in postoperative patients undergoing cholecystectomy conventional with nonlaparoscopic technique. The analgesic effect is not related to the inhibition of inflammatory mediator, interleukin 6 production.

## Figures and Tables

**Table 1:** Age, weight, and operative time between groups: expressed as mean values ± standard error of the mean. Analysis: KS test for normal distribution followed by unpaired *t*-test. *p* values <0.05 were considered significant.

	Control group	Tourniquet group
Age (years)	38.70 ± 3.422	40.80 ± 3.994
	*p* = 0.3472	

Weight (Kg)	64.60 ± 2.676	67.30 ± 3.337
	*p* = 0.2679	

Operative time (min.)	72.50 ± 3.819	67.00 ± 4.667
	*p* = 0.1869	

**Table 2:** Physical state as ASA. Data expressed as absolute numbers of patients in the respective ASA. *p* values <0.05 were considered significant. Analysis: Fisher's exact test.

	ASA I	ASA II
Control group	7	3
RIPC group	6	4
